# LoRa Traffic Generator Based on Software Defined Radio Technology for LoRa Modulation Orthogonality Analysis: Empirical and Experimental Evaluation

**DOI:** 10.3390/s20154123

**Published:** 2020-07-24

**Authors:** Alexandru Lavric, Adrian I. Petrariu, Eugen Coca, Valentin Popa

**Affiliations:** Computers, Electronics and Automation Department, Stefan cel Mare University of Suceava, 720229 Suceava, Romania; apetrariu@eed.usv.ro (A.I.P.); eugen.coca@usv.ro (E.C.); valentin@eed.usv.ro (V.P.)

**Keywords:** LoRaWAN, LoRa modulation, traffic generator, scalability, software defined radio

## Abstract

The digital revolution has changed the way we implement and use connected devices and systems by offering Internet communication capabilities to simple objects around us. The growth of information technologies, together with the concept of the Internet of Things (IoT), exponentially amplified the connectivity capabilities of devices. Up to this moment, the Long Range (LoRa) communication technology has been regarded as the perfect candidate, created to solve the issues of the IoT concept, such as scalability and the possibility of integrating a large number of sensors. The goal of this paper is to present an analysis of the communication collisions that occur through the evaluation of performance level in various scenarios for the LoRa technology. The first part addresses an empirical evaluation and the second part presents the development and validation of a LoRa traffic generator. The findings suggest that even if the packet payload increases, the communication resistance to interferences is not drastically affected, as one may expect. These results are analyzed by using a novel Software Defined Radio (SDR) technology LoRa traffic generator, that ensures a high-performance level in terms of generating a large LoRa traffic volume. Despite the use of orthogonal variable spreading factor technique, within the same communication channel, the collisions between LoRa packets may dramatically decrease the communication performance level.

## 1. Introduction

Over the last years, the number of IoT (Internet of Things) technologies has continually increased in our daily lives. The main goal of these technologies is to increase the quality of life. These IoT architectures have generated new challenges and issues, many of them being impossible to be anticipated at the moment of design. 

These challenges are mostly often related to: (1) Providinggreater device autonomy (mainly by obtaining the lowest possible energy consumption); (2) The possibility of integrating a high number of sensors; (3) Network scalability; (4) Providing a larger coverage area; and (5) Ensuring the costs are accessible to consumers targeted by this technology.

**Figure 1:**
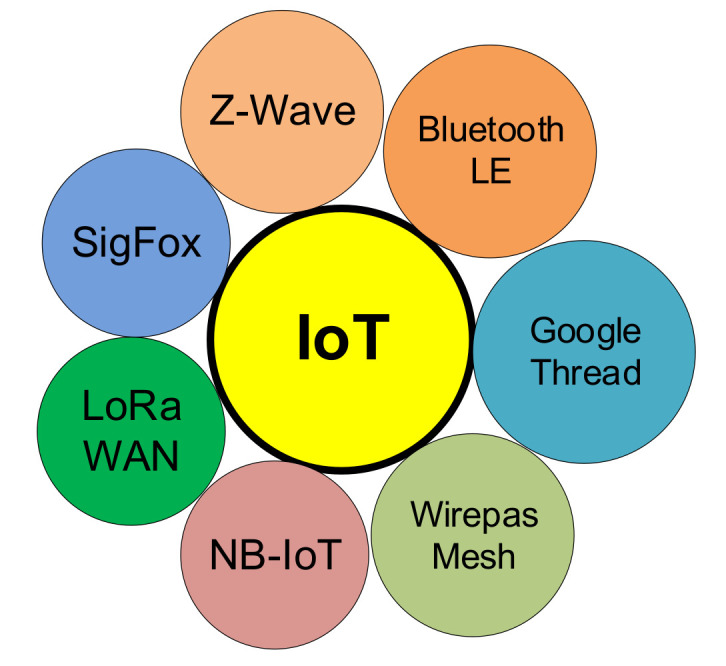
IoT communication protocols. Viable communication protocols that can be integrated into the Internet of Things (IoT) concept.

The main communication protocols that can be integrated into the IoT concept are presented in Figure 1. Amongst them are the following: SigFox [1], LoRaWAN (Long Range Wide Area Network) [2], NB-IoT (Narrow Band-Internet of Things) [3], Z-Wave [4], LinkLabs [5], Bluetooth LE (Low Energy) [6], Google Thread [7], and Wirepas Mesh [8]. One may consider these technologies to be the focus of the research community. The technologies can be separated by the following criteria: technologies operating in free frequency bands versus technologies using regulated frequency bands; technologies that are either open-source or vendor-locked; and technologies that use mesh versus star type network topologies.

Thus, in order to cope with the new challenges and IoT applications, LPWAN (Low-Power Wide-Area Network) networks have been created. A new communication paradigm for applications featuring low data rates with low power use has been achieved through the integration of technologies, such as SigFox or LoRa [1], with the goal of ensuring large coverage area, ranging from a few to as much as tenths of square kilometers. Out of the two technologies, SigFox is at a clear disadvantage by being vendor-locked—thus, its implementation is carried out by mobile network operators with the data transfer being done only by using SigFox cloud as middleware. 

The main protocols for solving the IoT concept challenge are briefly compared in Table 1. It can be observed that LoRa technology has more advantages that other proprietary technologies such as: Its open-source type that eliminates monthly subscription schemes, low cost of devices and great communication ranges going up to tenths of kilometers. The use of a star-type network topology means that each node can communicate directly with the gateway (GW) node, making for a simple and reliable network architecture. 

In this paper, we focus on the most prominent of the LPWAN (Low-Power Wide-Area Network) technologies, pioneered by Semtech, using the modulation called LoRa, the acronym for Long Range. The LoRa modulation technique scheme is an integrated part of the LoRaWAN [9] communication protocol defined by the LoRa Alliance. 

The main contribution of the paper is the evaluation of the orthogonality for the modulation of LoRa packets modulated with a different spreading factor within the same communication channel. Also, to further open new research directions regarding the scalability of LoRa networks a novel LoRa traffic generator able to emulate and simulate thousands of LoRa sensors without the need of further investments in a manner that is both low cost and effortless is designed and validated. From the authors’ best knowledge, an empirical approach backed by experimental results has not been covered yet in scientific literature, making for another novelty element of this paper. The ability to further test and develop new communication protocols, makes from the LoRa traffic generator an asset that can significantly contributeto large-scale LoRa networks research.

This paper has the following structure: First, the presentation of a short introduction explaining the goal of this work, it mentions the communication protocols that can be integrated into the IoT concept. The second section presents the LoRaWAN communication architecture. The third section presents the evaluation of LoRa modulation. This section provides a detailed assessment of performance in terms of Bit Error Rate (BER) when different spreading factors and communication channels bandwidths are used. The fourth section presents a novel LoRa traffic generator based on SDR (Software Defined Radio) technology. The LoRa traffic generator contribute to experimentally measuring the performance provided by LoRa under capture effect and the quasi-orthogonality condition of LoRa spreading factors. The fifth section makes for the conclusion of this work and the discussion of the obtained results. 

## 2. LoRaWAN Communication Architecture

The LoRa technology is part of the LPWAN family of technologies, especially employed when developing IoT networks, when the volume of data sent, oftentimes gathered from the sensors, is quite low (e.g., ranging from a few bits up to several hundreds of bytes). The operating range can reach up to tens of kilometers [10].

A proprietary modulation scheme is used by the LoRa communication, alongside a CSS (Chirp Spread Spectrum) type mechanism. The spread spectrum technology of LoRa modulation, when merged with digital signal processing technology, enables a very good performance, despite interferences [11]. 

Taking advantage of these mechanisms, LoRa communication can work over long distances in harsh environments. Different spreading factors are used, which provide different bit rates, providing an increase in the range of this communication model, leading to better spectral efficiency and an increase in network capacity. Physical bitrates are as it follows: They start from 11 kbits/s for SF7, and they go down to 250 bits/s for SF12 [12]. The LoRa communication uses unregulated frequency bands, with central frequencies of 433 MHz in Asia, 868 MHz in Europe and 915 MHz in the USA and Australia. 

**Figure 2:**
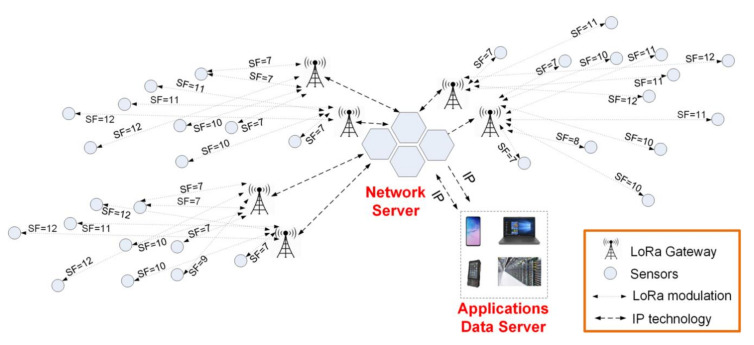
LoRaWAN architecture. It consists of sensors, gateways, a network server to allow user applications to access field data and a server for the application’s data.

The LoRaWAN communication system includes the following components: Sensors, also known as end-devices, a gateway to gather data and a network server, to allow user applications to access remote data with the help of an applications data server. The LoRaWAN communication architecture can be observed in Figure 2.

In Europe, limitations of transmission time are being regulated by the ETSI (European Telecommunications Standards Institute). Thus, a duty cycle of less or equal to 1% is allowed, that needs to be distributed by three channels of the EU sub-bands (868.1 MHz, 868.3 MHz, 868.5 MHz). There are three functionality classes for the end-devices: Class A, B and C. Most LoRaWAN devices have a Class A rating, with transmission being initialized by the end-device node, in a completely asynchronous manner. After the end-device node sends data to the gateway (uplink), two well-defined time slots are allocated to it during in which a response from the gateway is expected (downlink). The first of these time slots is opened in the standard frequency band of 868 MHz, using the same channels as the uplink ones. The second time slot is opened in a reserved frequency band (869.525 MHz). 

This method increases data packets reception probability and the immunity to interferences. These interferences can originate from other signals transmitted in the same network, on the same frequency band when the network is clustered with too many sensors that send the information to the gateway. 

Class B devices must be compatible with Class A devices. Despite being very similar to Class A devices, Class B devices require an additional well-defined time slots, where a message from the gateway is expected. In this case, the sensor will receive a time-synchronized beacon-type signal from the gateway, to tell exactly when the node is in receiving mode. The largest energy consumers are Class C devices, since they shift to the reception mode after sending data to the gateway. Moreover, these Class C devices must be compatible with Class A devices only. 

Unlike other LPWAN wireless transmission technologies, LoRa uses the same phase between two chirp symbols for the preamble of the data packet at the physical layer of the communication protocol. This mechanism leads to improved time and frequency synchronizations, without the need of using expensive hardware elements as decoders in the configuration of the end-device node.

## 3. LoRa Modulation Evaluation

In this section, we focus our attention onto the RF (Radio Frequency) and MAC (Medium Access Control) layer particular to the LoRa technology.

Current studies are mostly based on the results obtained through simulations [13,14], and imply that the use of multiple transmission channels and spreading factors lead to a system that can be deemed as a superposition of independent sub-systems (featuring one channel, with one single SF) [15]. This is only a powerful simplification, especially when the SFs adopted by LoRa are quasi-orthogonal [16]; and as a consequence, in certain conditions, the collisions can prevent the correct reception of overlapped transmissions by using different SFs. In Reference [17] the power reception thresholds are quantified for various SF and the signal to interface ratio (SIR). Despite all these, there is a justification of the obtained results in the work mentioned before. Thus, the purpose of this work is to provide the readers with a series of clarifications and sets out to analyze the influence of collisions onto the performance level of a LoRa network and the orthogonality of LoRa communication [18]. 

In this work, the empirical results are justified by the obtained experimental results. The influence of collisions over performance is difficult to analyze in a classical setup, due to the fact that the integration of a large number of sensors demands a high cost. This is the reason we focus on the development, implementation and testing of a LoRa traffic generator that allows us to generate a large volume of data packets with the use of SDR technologies. 

This LoRa traffic generator must be easy to re-configure and flexible enough to allow for the customization of LoRa packets enabling testing in various configurations and structures. Because of these reasons, an SDR solution has been selected, meeting the requirements of flexibility, while being low cost at the same time. The traffic generator tests are performed within a semi-anechoic chamber in order to avoid any outside interference and obtain accurate results. 

The performance testing of the LoRa technology cannot be overlooked. The developers and those who implement networks which use the LoRa modulation need new solutions that can contribute to the direct increase in performance level meaning a detailed analysis of the influence the collision of packets, which can occur in LoRa communication architectures. These aspects can be considered the novelty of this work. In Reference [17], it is shown that the SIR threshold for the reception of a correct packet is almost independent of SF, with a channel rejection average of −16dB.

The goal of introducing these separation systems (e.g., spreading factor and communication channel bandwidth) is within their possibility to integrate several nodes that are as high as possible capable of co-existing within the same cell provided by a gateway (GW) module. On these grounds, LoRa offers various possibilities for orthogonal transmissions, such as central channel frequency (f_c_) Spreading Factor (SF), communication channel bandwidth (BW) and Coding Rate (CR). Notwithstanding all this, despite the ruggedness of the LoRa technology, its scalability is still undergoing research with a collective effort aimed at achieving this goal [19]. This paper comes to fill this gap in scientific literature, contributing to the opening of new research directions in the field of LoRa network scalability. 

In a system that uses LoRa modulation, a collision is described as a moment in which two or more sensors simultaneously send data to each other by using the same spreading factor on the same channel. These collisions could lead to the loss of network packets through its action. Despite all this, thanks to its capture effect, the receiver can sometimes successfully decode one of the packets even when a collision may happen [20]. The capture effect can be defined as the capacity of the receiver to decode a packet even when a collision between multiple packets occurs (usually the packet with the higher Signal-to-Noise Ratio is decoded).

The spreading factors and settings, such as the bandwidth of the LoRa communication channel allow the theoretical orthogonal transmissions between the LoRa sensors; this aspect can prevent interferences between networks, but those mechanisms require further in-depth study and analysis. Concomitantly, the evaluation of the orthogonality of combinations between the spreading factor and communication channel bandwidth is analyzed in this work. Despite all this, the evaluation of performance level when it comes to simultaneous communications with the same or a different SF within the same LoRa communication channel represents a notable challenge. Furthermore, when a traffic increase occurs, this avalanche collision effect (particular to ALOHA network types characteristic of LoRa networks) triggers a decrease in performance level [21,22].

The LoRa specification, as defined by Semtech, has seven spreading factors (SF6–SF12) and four different channel bandwidths (62.5 kHz, 125 kHz, 250 kHz and 500 kHz) following Semtech-defined LoRa specifications. In practice and current hardware implementations, SF can be varied from 7 to 12 with the employed BWs being 125 kHz, 250 kHz and 500 kHz. As per channel allocation possibilities are specified by one of the IoT platform named TTN (The Things Network) [23], there are a number of 9 channels that are defined in the EU zone within the SRD860 unlicensed frequency band that make the uplink operation possible. By the same specification, the 250 kHz channel bandwidth can be used only within channel number 2 with a central frequency of 868.3 MHz. Channel number 9 (with a central frequency of 868.8 MHz) is used for high-speed transmission and uses FSK (Frequency-Shift-Keying) modulation.

Between specific spreading factors and various bandwidths, theoretical orthogonality and more importantly, non-orthogonality can be easily determined. Not all combinations of SF and BW are orthogonal [24]. Theoretically, if the same SF is used on the same communication channel, the level of performance decreases dramatically, as the orthogonality conditions are not met [25]. 

The LoRa communication model presented in Reference [26] has been integrated and modified in the present analysis. The initial model could only generate LoRa packets with a payload of 20 bytes. Due to the characteristics particular to the LoRa communication model, it is very probable that when two signals both modulated with the same spreading factor collide, the strongest of these signals will be correctly demodulated, due to the capture effect. The main drawback of the initial model [26] is in the lack of a modification option for the payload of the packet. Another drawback is the fact that the user cannot change the bandwidth of the communication channel. Considering those drawbacks, the first part of this paper presents the improvements in the model. 

Thus, the initial model [17], has been improved with the following additions: The packet payload customization, bandwidth of the communication channel and the central frequency of the communication channel selection. These improvements grant the model a high degree of adaptability, creating a powerful instrument in the evaluation of LoRa scalability. 

The modulated packet is then interfered in turn with a random LoRa packet modulated by a spreading factor, so that all possible variations are analyzed. The interfering packet is sent at random moments in time with an SIR (Signal-to-Interference Ratio) which increases by a 1 dB step starting from −30 dB. By implementing this mechanism, the BER (Bit Error Rate) characteristic can be determined, as a result of comparing later demodulated bits to initial modulated ones. It can be concluded that, the lower the spreading factor that interferes with the communication, the higher the SIR threshold necessary to obtain an admissible BER value. The model is implemented using MATLAB [27] environment and allows the modulation and demodulation of LoRa packets without any in-band noise from other interference sources. 

With the integration of the possibility of change for the bandwidth of the communication channel and applying the model on different LoRa packets featuring different payloads, the conclusion is that the applicability of the model is greatly extended. Thus, the new model offers the possibility of analysis of the influence of the communication channel bandwidth and of the payload dimension over the level of performance of the LoRa technology, by determining its scalability through the evaluation of the BER parameter. 

As expected, the worst-case scenario corresponds to when the packet interferes with a packet modulated with the same spreading factor. The limitations of this approach are related to the fact that an empirical method is used, and some limitations of the hardware design of the sensors and of the gateway modules are not taken into account. Moreover, it is important to mention that the assumption of perfect synchronization between transmitter and receiver is considered. 

Another major difference is in the fact that the LoRa traffic generator that has been developed and tested uses a method that allows the customization of generated packets with an innovative solution, unlike other approaches like the one presented in Reference [26]. Different approaches that are presented in scientific literature make use of a capture technique of a packet sent by a LoRa sensor. This means that the packet is later simply retransmitted. The disadvantage of this approach is the limitation of user options, due to the impossibility of changing the LoRa data packet characteristics. Arguably, the empirical analysis backed by an experimental evaluation represents the main contribution of this work. These results open new directions of research when it comes to sensor networks that use LoRa modulation.

Thus, two overlapped transmissions have been considered by a different use of spreading factors in order to evaluate the performance level. Thus, each packet modulated with an SF is overlapped (the observed packets are transmitted on the same channel at random time intervals) with a packet that has been progressively modulated with all other possible SF combinations. This is the emulated communication scenario. Following the implemented model, the correct synchronization between the sensor and GW is implied, the interference packet is randomly generated in time. All these elements make the degradation in performance of maximum importance.

**Figure 3:**
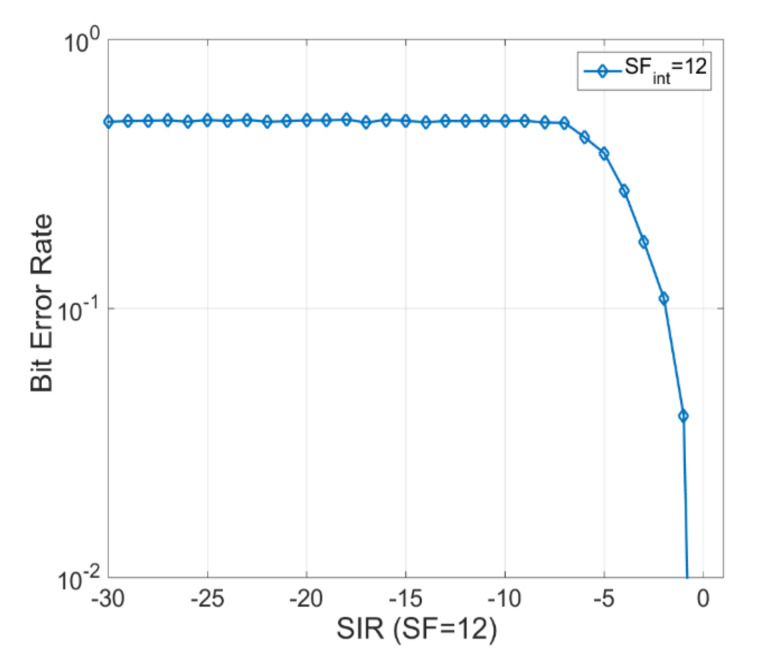
Bit Error Rate (BER) parameter for SF12, bandwidth (BW) = 125 kHz. The figure depicts the BER parameter when the SF12 spreading factor is used for a 125 kHz channel bandwidth. One can observe a decrease in performance.

Figure 3 depicts the BER parameter when the SF12 spreading factor is used (the most unfavorable case corresponding to the situation when the transmission duration of the packet is maximum being of the order of seconds). In this situation, a 125 kHz bandwidth is used. One can see that at the same time as the SIR (Signal-to-Interference ratio) parameter decreases, the performance level of the LoRa communication suffers a sharp drop. 

The SIR parameter has been chosen to evaluate performance as accurately as possible, considering the LoRa communication channel. One can observe that for an SIR value of approximative −1 dB, the BER value equals to about 70%. There is still the need to mention the fact that the most unfavorable case is analyzed when all sensors use the same SF12 spreading factor.

Moreover, the spreading factor has been modified from SF12, representing the most unfavorable case (with the sensor located at a great distance from the GW) to SF6 corresponding the situation in which the sensor is located at a short distance from the GW. For each analyzed SF, every spreading factor is taken into consideration as possible interference. Obviously, the maximum value is reached when all the other sensors communicate on the same SF as the one undergoing analysis; this situation is also what we refer to as the worst-case scenario. This is a pessimistic approach and represents the evaluation of a boundary situation which is, however, often met with during the practical implementations of the LoRa technology. Due to the fact that one of the main advantages of the LoRa technology consists in large communication distances of the order of tens of kilometers; the operators of these networks will choose an SF with the maximum value available and implicitly ensuring communication at long distances, from an incentive to minimize the number of gateways (e.g., because of cost) which serves a certain area. 

**Figure 4:**
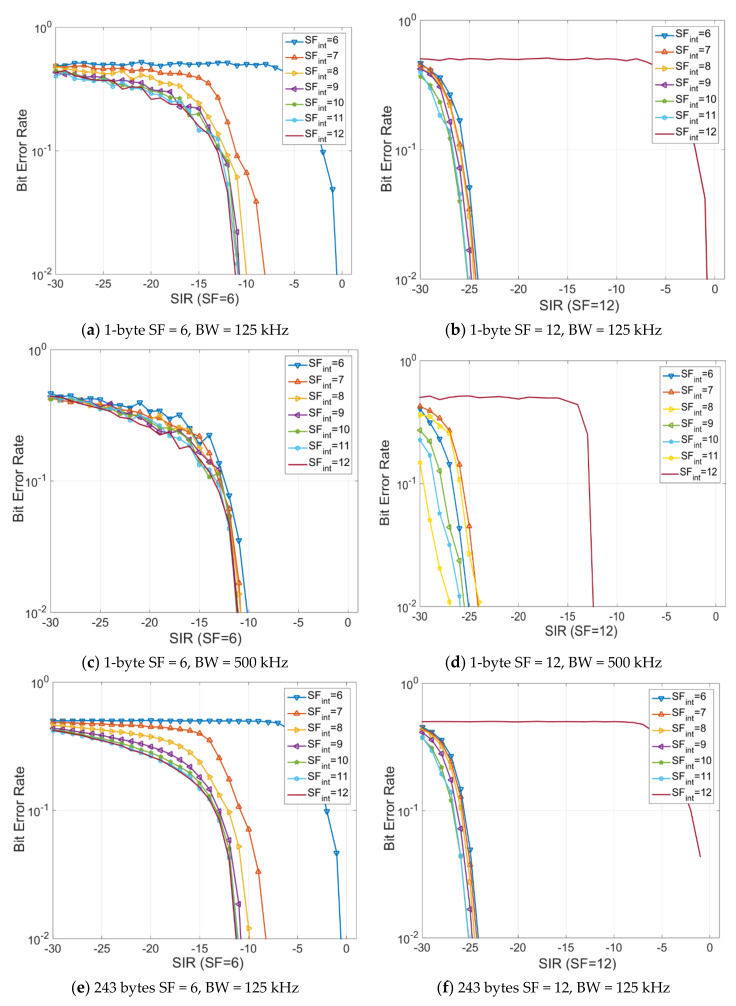

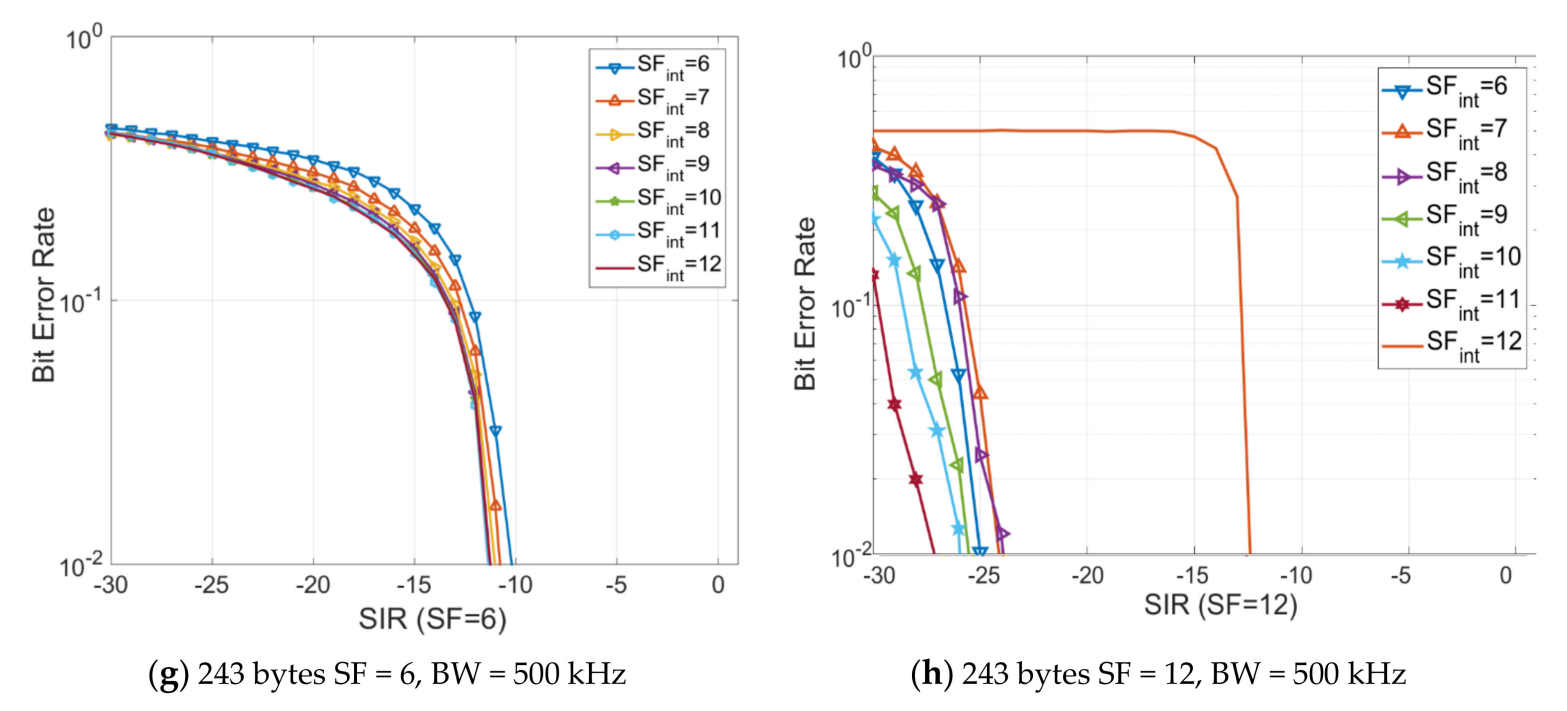
The analysis of performance based on SF, BW and packet payload variation. The SF is varied from SF6 to SF12 and the bandwidths analyzed are 125 kHz and 500 kHz, respectively. In the evaluation scenarios, the payload is varied from 1 byte to 243 bytes.

This feature determines a longer transmission time (due to the LoRa modulation mechanism) and implicitly the increase of probability of a collision occurring [28]. The results of these simulation scenarios are depicted in Figure 4.

Thus, another varied parameter is the payload size associated with the LoRa packet ranging from the minimum value of 1 byte to a maximum LoRa specification defined value of 243 bytes. From the obtained results, it can be observed that with SIR (Signal to Interference Ratio) decrease the analyzed BER (Bit Error Rate) parameter increases, an expected aspect due to the increase of the transmission-related time. When a larger channel bandwidth is used, the performance level and the resistance to interferences is considerably enhanced.

Furthermore, one can see that once the packet payload is increased, the resistance to interferences is not drastically affected, with results being similar. Since the studied scenario is of a worst-case type (e.g., use of SF12) and of best-case scenario (e.g., use of SF6), the payload influence is not a determining factor in the final overall result. This result is a surprising one, due to the fact that the answer to a wireless communication channel is oftentimes dependent on network packet dimension. A similar result has been reported in Reference [29]. Thanks to the coding and spectrum spreading mechanism patented by Semtech, the influence of payload over the level of performance is minimized. In the eventuality in which the receiver and the transmitter are synchronized, the influence of payload over performance level is minimal when talking about co-existence. 

**Figure 5:**
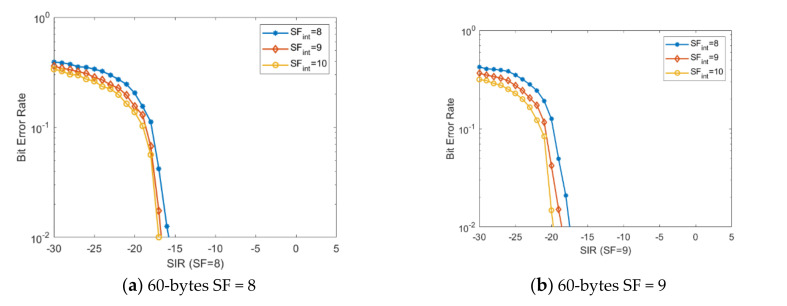

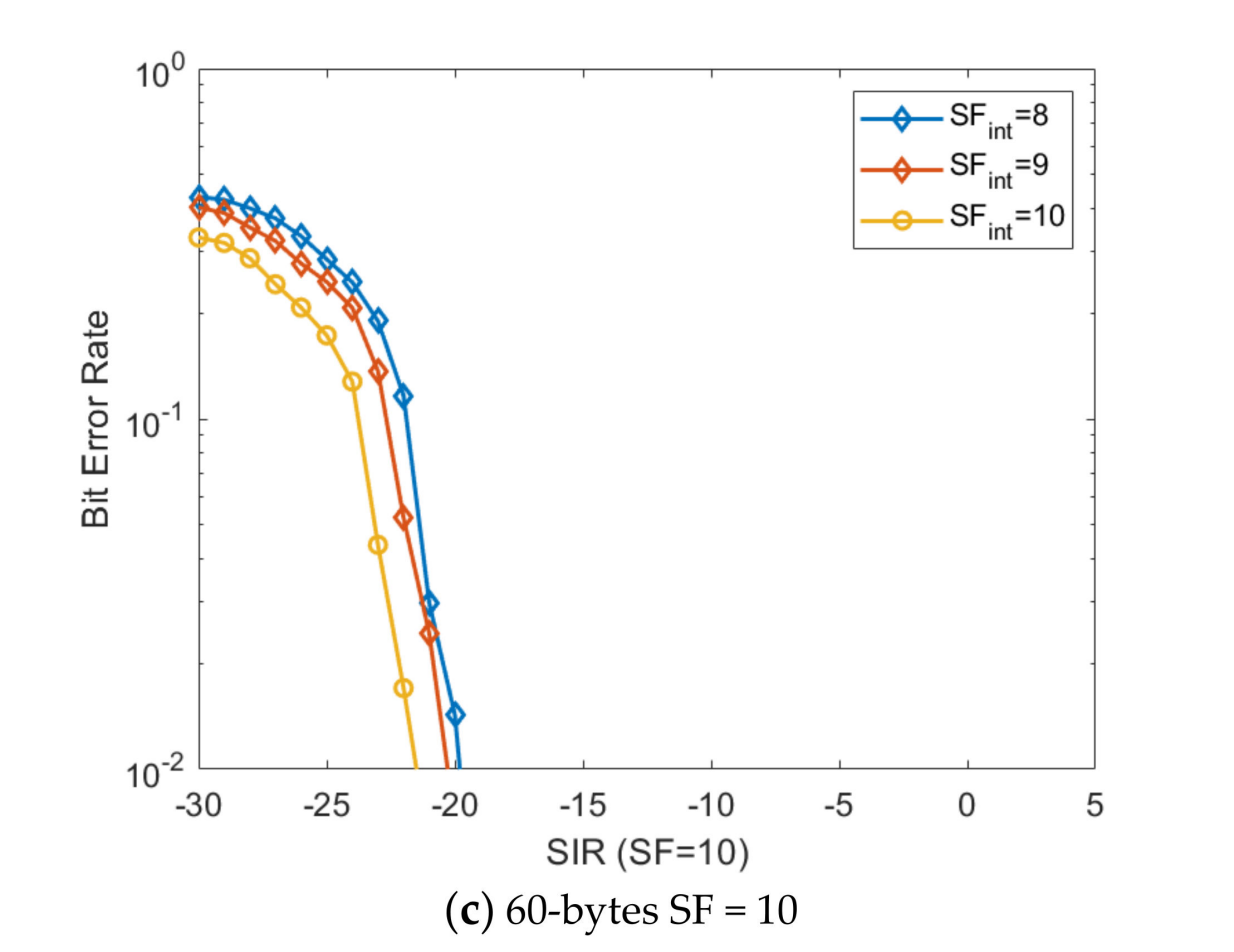
The analysis of performance for SF8 when using a packet payload of 60 bytes. The SF is varied from SF8 to SF10, and the analyzed bandwidth is 500 kHz.

Figure 5 presents the performance analysis for SF8 when using a packet payload of 60 bytes. The SF is varied from SF8 to SF10, and the analyzed communication channel bandwidth is 500 kHz. This scenario was included in order to create a more accurate overview of the impact of SF selection for LoRa sensors on the performance level of the communication link, since the previous simulation scenarios were focused on the most favorable and unfavorable use cases.

**Figure 6:**
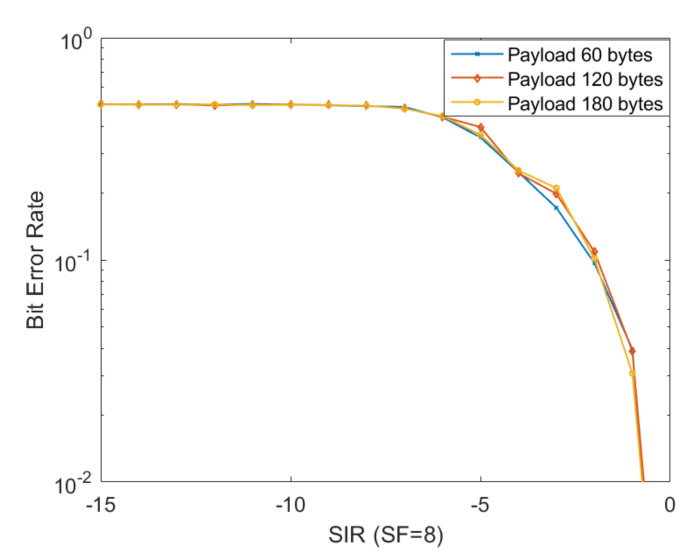
BER obtained by varying the payload size while maintaining the same BW value. The payload was varied from 60 bytes to 120 bytes and 180 bytes.

To investigate the influence of the payload on the resistance to interferences, another simulation scenario involving the BER parameter evaluation for a specific SF by varying the payload size while maintaining the same BW value was performed. The payload was varied from 60 bytes to 120 bytes and 180 bytes, respectively. The selected communication channel bandwidth in this setup is 125 kHz. In Figure 6, we can observe the obtained results.

As stated before, one can observe the payload does not significantly influence the performance level of communication. We consider this payload analysis to be one of the main contributions of this work. This aspect is particularly important for LoRa network developers that can consider the use of large packets if the specifications of the application allows it.

**Figure 7:**
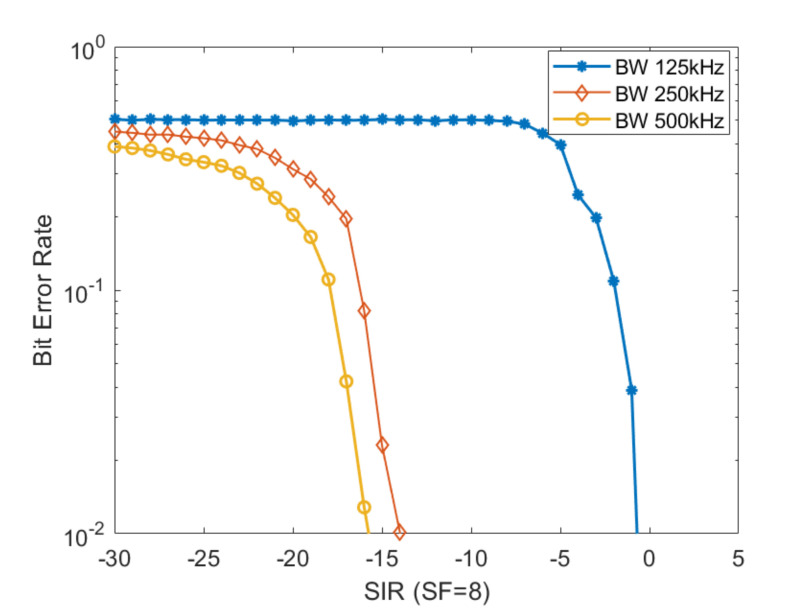
BER curves for a specific SF obtained by varying the BW size from 125 kHz to 250 kHz and 500 kHz, respectively, while maintaining the same payload size 120 bytes.

In order to evaluate the influence of the communication channel bandwidth on the level of performance, an additional test had been performed. We measured the BER parameter by varying the bandwidth of the communication channel, while maintaining the same packet payload size of 120 bytes. These results are presented in Figure 7. The goal of these assessments was to analyze the influence of the communication channel bandwidth on the performance level of the communication link.

Our results show, as expected, that with the BW increase the performance level of the communication significantly increases. Thus, a larger communication channel bandwidth is preferred, if available, however, but it is dependent on the geographical area of the installation site.

From the presented results, it can be seen the collision number contributes to the dramatically decrease in the performance level of the LoRa communication. Thus, this aspect, which cannot be overlooked, needs to be carefully addressed and further analyzed when a high level of performance for a high-density large-scale type network is mandatory [30].

## 4. LoRa Traffic Generator Development and Validation

**Figure 8:**
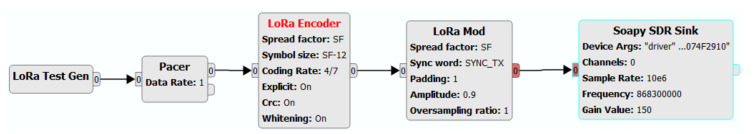
LoRa traffic generator by integrating Software Defined Radio (SDR) technology. The integrated communication mechanism can generate a high volume of data with the help of LoRa packet transmissions.

In this section, an experimental analysis has been made using SDR technologies , measurements being realized in a semi-anechoic chamber (in an interference-free environment). while considering the previously presented results. The first step has been the development, implementation and testing of a LoRa packet generator capable of generating packets that are compatible with the LoRaWAN specifications which have been defined by LoRa Alliance. For the implementation of the generator, the SDR technology is integrated by means of a LimeSDR [31] hardware equipment. Figure 8 depicts the developed communication mechanism. This advantage can be achieved by integrating two or more SDR platforms, each equipped with two transceivers in a topology that is of a MIMO (Multiple Input Multiple Output) type. Thus, there is a possibility of generating a large LoRa traffic volume with an increase in performance level. The advantages of this implementation are represent ted by the fact that the user does have access to the mechanism tasked with generating the packet, such as payload or elements that are tied to the ways of coding and creating packets. These features represent the novelty of this work.

To validate the developed LoRa traffic generator, some test scenarios created by means of using a TDK RF Solution [32] manufactured semi-anechoic chamber has been implemented. This is because an isolated environment is required with no in-band interferences, especially when analyzing an unlicensed frequency band where the transmitted signal level is very low (below RF noise level).

**Figure 9:**
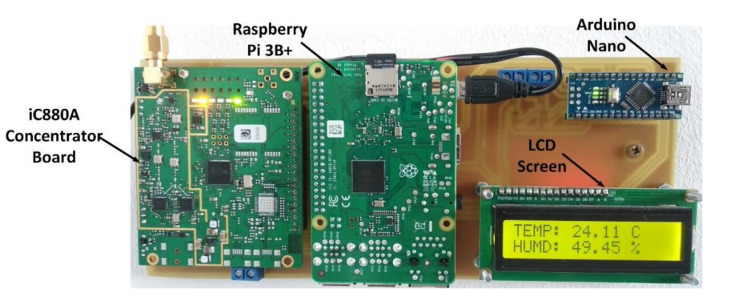
LoRaWAN gateway. The gateway integrates a Raspberry PI 3B+ and the iC880A concentrator that allows the simultaneous communication on eight channels (as stated in the LoRaWAN specifications).

The used GW is implemented by using a Raspberry PI 3B+ and the iC880A concentrator board running a Linux-based operating system and integrating a forward-packet mechanism type towards the TTN data aggregation platform, connected to the Internet inside a semi-anechoic chamber. The designing and testing of the GW module are depicted in previous work published by the authors [33]. The GW structure used in the conducted experiments is presented in Figure 9.

**Figure 10:**
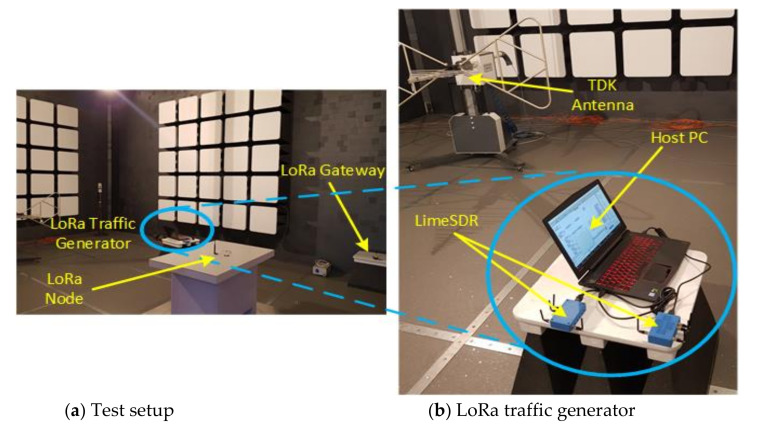

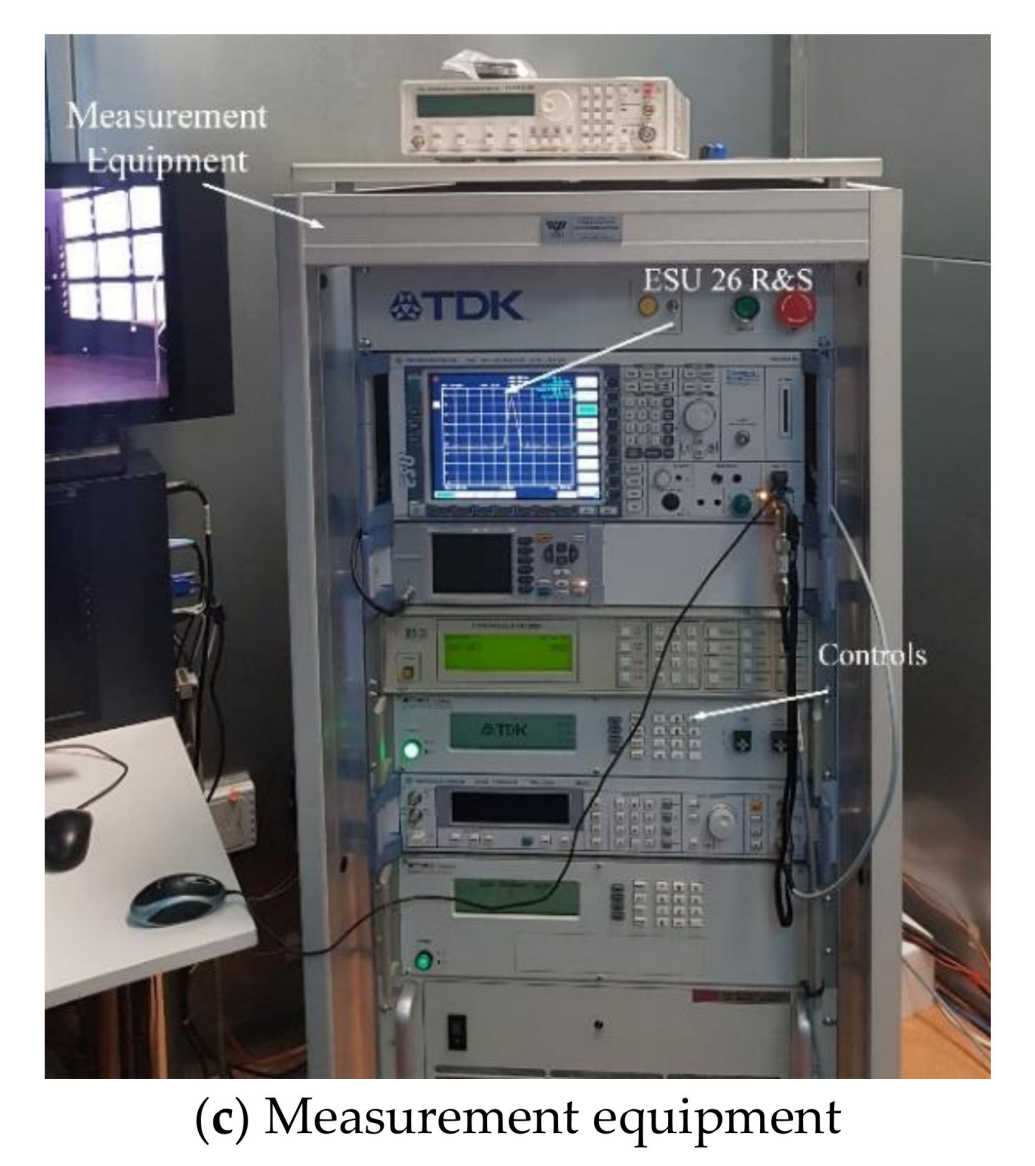
Performance evaluation test setup. (**a**) presents the test setup configuration. (**b**) depicts the traffic generator that has been realized by using two SDRs of a LimeSDR type and the host PC, which runs the application. (**c**) presents the measurement equipment.

Figure 10 depicts the test setup implemented in the evaluation of the performance level of the LoRa traffic generator module so that the resistance to interferences that originate from the use of different SFs can be evaluated. The test makes use of a TDK HLP-3003C periodic log antenna. 

In the experiments carried out, the LoRa sensors that have been used feature an Atmega328 micro-controller and an RFM95 [34] radio module from HopeRF. These sensors communicate with the GW module by means of different SFs, and as a communication channel, 868.3 MHz is used. For the conducted tests, the payload has been set to 12 bytes, due to considering this the average dimension of a LoRa packet [35]. In these experiments, a handful of test scenarios has been performed for the validation of the developed traffic generator.

The main advantages are that the user has access in modifying parameters such as: Spreading factor, a packet payload, coding rate, the communication channel bandwidth and the communication channel central frequency. The second advantage of the traffic generator is to generate a very large number of LoRa packets so that it can allow for the emulation of LoRa networks of a high-density large-scale type and by doing so effectively opening new research directions within the field of wireless sensor networks.

Thus, the architecture that has been initially presented (e.g., by means of a single SDR platform) has been developed and perfected with the introduction of additional multiplexers with a large data volume of generated traffic as a result. 

**Figure 11:**
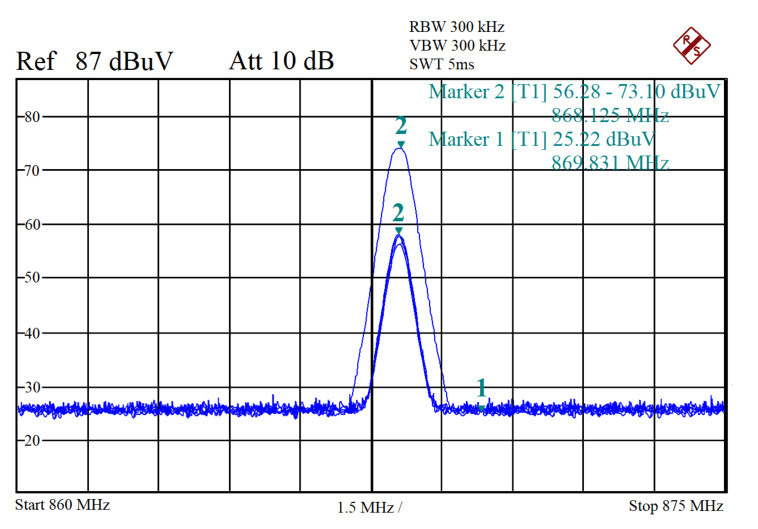
LoRa traffic generator, spectrum. It can be observed that the LoRa sensors communicate on the same channel as the LoRa traffic generator. Thus, the LoRa traffic generator can obtain LoRa concurrent transmissions so that a high number (e.g., thousands) of nodes can be emulated.

Figure 11 depicts the packet spectrum of various power levels taken from within the semi-anechoic chamber. The LoRa traffic generator is able to generate data from thousands of sensors, which makes it a powerful instrument for performance assessment and scalability evaluation. 

Due to its low cost it can be easily implemented by researchers in a low effort manner. Another advantage is its scalability, by simply integrating other SDR devices the traffic generation capacity may be proportionally increased. 

**Figure 12:**
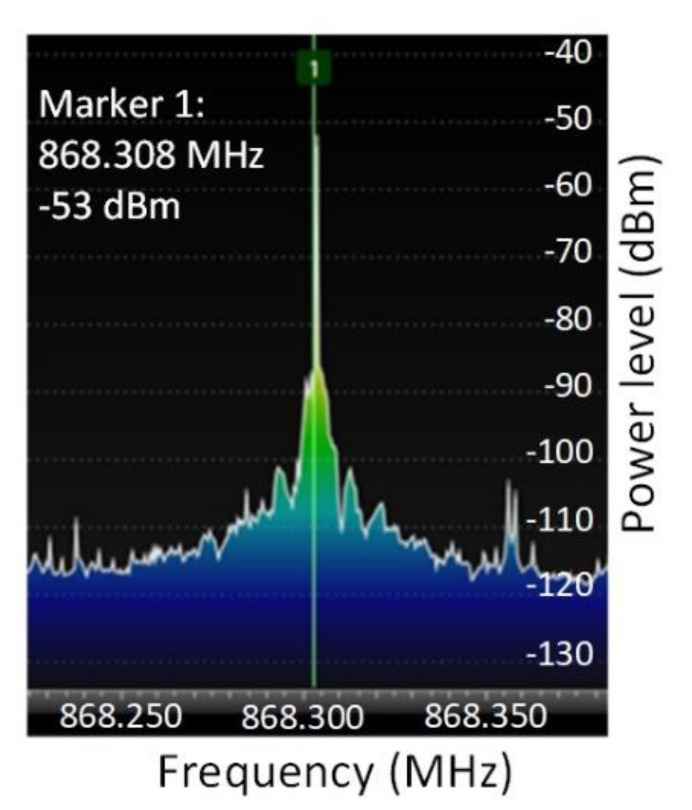
LoRa packet spectrum. The packet created by the LoRa traffic generator respects the LoRaWAN specifications and is received correctly by the gateway module.

Figure 12 depicts the spectrogram of a LoRa packet by using the architecture mentioned in Figure 5. The central communication channel frequency has been of 868.3 MHz. This implementation variant ensures a high level of performance. As seen, all the packet characteristics respect the LoRaWAN specifications.

The validation of the proposed traffic generator has been done in an evaluation scenario, where the spreading factor has been modified from SF7 to SF12, alongside the channel bandwidth, from 125 kHz to 250 kHz, only for SF7.

**Figure 13:**
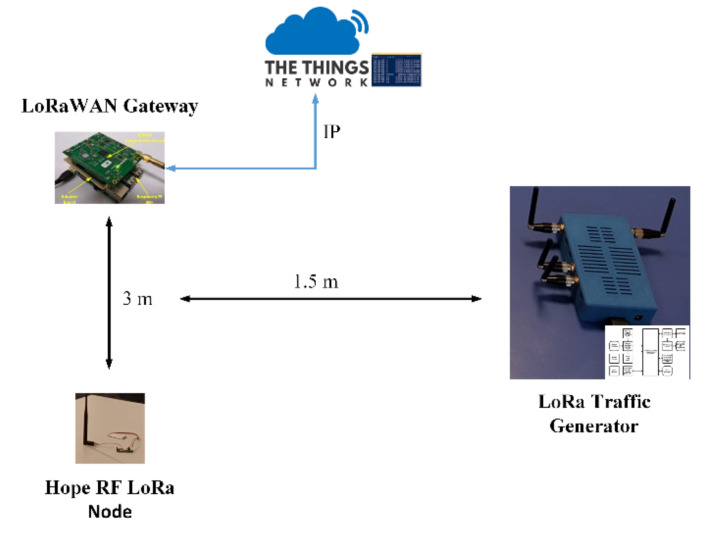
LoRa packet spectrum.

Figure 13 presents the performed evaluation scenario setup in the anechoic chamber. As previously stated, this includes the Hope RF LoRa node, the developed LoRa gateway that is connected to the TNN platform and the implemented SDR LoRa traffic generator.

It has to be mentioned, that all of the packets are correctly received by the GW when the LoRa traffic generator is not active. The LoRa traffic generator and the sensors use the same transmission power level. Thus, when the traffic generator is used, there is a collision that occurs between the packets sent by the LoRa generator and the packets sent on the same channel by the sensor. 

In the performed tests, the LoRa traffic generator acts as a source of interference. The monitored communication link from a performance standpoint belongs to the LoRa sensor node that uses an Atmega328 micro-controller integrating an RFM95 radio transceiver. The sensor communicates with the GW module by means of different SFs, on 868.3 MHz communication channel and generates one packet every two seconds. The received packets are sent by the GW module to the TTN platform. The LoRa sensor node can use one SF at a time, which can be modified by changing the software. 

The SDR LoRa traffic generator varies the SF parameter of the transmitted packet generating packets at random time moments using the same 868.3 MHz communication channel. These packets are received by the GW module and are reported to the TTN platform. The MIMO structure of the SDR platform allows the architecture to send different packets with different modulated SFs. Each transmitter can send packets modulated with a different SF. In the performed tests the payload was varied from 1 byte to 243 bytes. As the payload increases, the performance level is not affected, like the results shown in Section 3, obtained in an empirical simulation level. 

**Figure 14:**
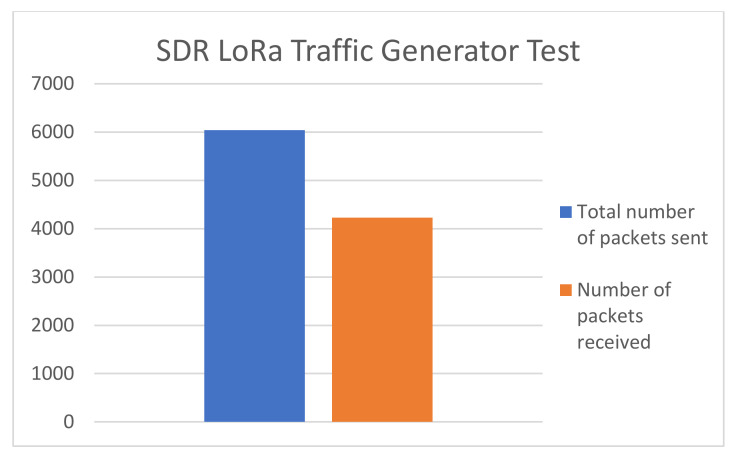
LoRa traffic generator results.

The LoRa traffic generator results are depicted in Figure 14. A total number of 6043 packets were sent on 868.3 MHz communication channel. Out of all the gathered results of the experiment, only approximately 4230 were received and recorded by the GW. We can conclude that approximately 30% were lost, due to collisions occurring at the receiving side. Upon closer inspection, it is revealed that the packets that have been lost are precisely the ones which have been sent at the exact same time and used the same SF notwithstanding payload dimensions. 

We need to mention that this evaluation setup corresponds to a pessimistic scenario that does not consider the geographical duty-cycle restriction that shows that a node must respect amongst many other node class restrictions. The emulated scenario corresponds to a high-density LoRa communication architecture that integrated thousands of nodes.

The simulated results presented in Section 3 do not take into account the constraints in the hardware implementation of the LoRa devices. The simulated results are empirically generated and do not consider the hardware implementation limitations and assume that the transmitter and receiver are synchronized. 

Once an experimental setup is implemented, all these assumptions are no longer valid, since the hardware implementation of the GW module and of the LoRa sensor lead to results that differ from those determined on an empiric basis. The scenario that involves the generation of a high-volume data packets by the LoRa traffic generator is a pessimistic one, due to the fact that it takes into consideration a boundary condition by tackling the duty-cycle restrictions associated with the LoRa communication in some geographical areas. The synchronization between the LoRa sensor and the GW module is crucial for the correct demodulation of the received packets [36]. The action of generating packets by the LoRa traffic generator determines the apparition of collisions that lead to some of those packets not being received by the GW module. Thus, performance drastically drops. The synchronization between receiver and transmitter is done by eight preamble symbols with the packet not being received when these symbols are corrupted, due to a collision [37]. Based on this observation, the sharp loss in performance in a LoRa communication architecture is caused by the occurred collisions. 

However, in a high-density large-scale type of communication architectures (where sensors are spread across a large geographical area), this pessimistic analyzed scenario corresponds to a scenario of mass-deployment. These analyses are necessary for the evaluation of scalability of the LoRa technology, so that whoever implements and develops such type of LoRa networks can take into account aspects that show the possibility of eventual collisions occurring with performance loss as a direct result. 

## 5. Conclusions

From a theoretical standpoint, the use of different spreading factors at different channel bandwidths onto the same communication channel can warrant orthogonality. However, the empirical and practical results show that collisions do occur, and that they drastically decrease the level of performance. The simulation results are validated with the implementation of a novel LoRa traffic generator by using SDR technology that ensures a high level of performance. The advantage of a traffic generator is represented by the possibility of emulating different LoRa sensor networks, without the need for additional costs in what can be described as an effortless and cheap manner. This aspect opens new research directions in the field of sensor networks by addressing the various issues which apply to the IoT concept. From the empirically obtained results, it can be seen that despite the existence of orthogonality between different spreading factors within the same communication channel, the collisions between certain LoRa packets may occur (especially in the case of far communication links). This aspect cannot be overlooked and must be further analyzed with it being of utmost importance to developers and users who implement communication networks of a LoRa type.

Another interesting result is that the payload influence is not a determining factor regarding the resistance to interferences. Thanks to the coding and spectrum spreading mechanism patented by Semtech, the influence of payload over the level of performance is minimal. If a larger channel bandwidth is used, the performance level and the resistance to interferences is considerably enhanced.

These results have important implications for the LoRa operators and network planning professionals: Allotting larger SFs for users farther away would not necessarily improve the connectivity capability in high-density large scale sensor networks, since the transmissions are later received at low-power levels and are very prone to collisions due to longer transmission times.

Moreover, if a CCA (Clear Channel Assessment) mechanism is not integrated, the ALOHA communication mechanism can lead to lowered performance. In scientific literature, there are some works that propose the integration of different control mechanisms for the communication channel assessment, such as the Listen Before Talk mechanism which significantly increases the performance of the LoRa communication system [38,39]. 

As the LoRa communication network increases (e.g., integrates thousands of sensors), a real decrease in the number of collisions occurs, once a Listen Before Talk mechanism, based on a channel energy-detection procedure has been integrated at a physical layer of the communication protocol [40]. The second option worthy of consideration is the implementation of a CCA type of mechanism at MAC communication layer. The results show that by integrating such mechanisms in a LoRa network, there is a possibility of increasing the number of integrated sensors. 

From the author’s standpoint, at the moment, there is a requirement to find certain solutions which can contribute to the increase in the performance level of LoRa networks. From the obtained results at a simulation level, as well as the results obtained by experimental means within the LoRa communication collision, can occur if two or more LoRa packets are received on the same channel. Despite the advantages of the LoRa technology, namely, its ruggedness (e.g., resistance to interferences) and long communication range, its scalability is still undergoing research. This is a goal shared by the scientific community. 

At the moment, there are several adaptations of the LoRa protocol that allow its use in industrial applications with the help of some solutions that overcome the ALOHA communication scheme. Certain works promote the use of a time-slot schedule to reduce and eliminate the number of collisions and to allow the grouping of sensors present in certain areas based on the level of the received signal. In such conditions, one sensor can only communicate within the programmed time slot. The Listen Before Talk contention-based protocol verifies the degree of channel occupancy multiple times within the same scheduled time slot [41]. By integrating such a mechanism, the decrease in the latency level associated with the LoRa communication is possible alongside with the decrease in the number of collisions. 

Another goal is the improvement of the LoRa communication protocol, so that it becomes suitable for real-time environments and time-sensitive applications. One other method of nearing the LoRa communication protocol to the real-time concept involves the introduction of the ability of self-management and grouping of the devices, so that they themselves can determine their position on the communication time slots, as part of an architecture that is based on time-slotted communications. The use of a single slot dedicated to each frame is suggested in Reference [42] as a solution for global synchronization and for the management of acknowledgements. The experimental results show that the implementation of such mechanisms leads to a packet delivery rate of over 99%, with an added plus of energy efficiency, even in the case of distant sensors. 

The novel LoRa traffic generator that has been developed is a useful and reliable instrument for the analysis of scalability and performance of LoRa communications. The traffic generator can be integrated into many applications, having immense potential. Thus, different manufacturers can use it in assessing the real throughput of their gateway architecture. Moreover, LoRa network operators can easily field-test their network distribution on a large-scale geographical area, reducing the initial deployment costs. The ability to further test and develop new communication protocols makes the LoRa traffic generator an asset that can provide a contribution to large-scale LoRa networks research.

## Figures and Tables

**Table 1:** Comparison of IoT communication protocols.

Parameters	NB-IoT	SigFox	LoRa	Google Thread	WirePas Mesh
Channel Bandwidth	180 kHz	100 Hz	125 kHz	600 kHz–5 MHz	2 MHz
Full Duplex	No	No	No	No	Yes
Downlink	127 kbps	100–600 bps	300 bps–50 kbps	20–250 kbps	1 Mbps
Uplink	159 kbps	100–600 bps	300 bps–50 kbps	20–250 kbps	1 Mbps
Latency	<10 s	>20 s	1–2 s	<100 ms, 1 s	<10 ms
Data Limit	NA	140 packets/day uplink, 4 packets/day downlink	NA	NA	NA
Duty Cycle	NA	Max 30 s/h (<1%)	<1%	<1% in some frequency bands	NA
Allocated Bandwidth EU	200 kHz	200 kHz	125 kHz	-	-
Maximum Transmission Power	14, 20, 23 dBm	22 dBm	22 dBm	0 dBm	<10 dBm EU
